# Five-day rehabilitation of patients undergoing total knee arthroplasty using an end-effector gait robot as a neuromodulation blending tool for deafferentation, weight offloading and stereotyped movement: Interim analysis

**DOI:** 10.1371/journal.pone.0241117

**Published:** 2020-12-16

**Authors:** Kyo-in Koo, Chang Ho Hwang

**Affiliations:** 1 Department of Biomedical Engineering, School of Electrical Engineering, University of Ulsan, Ulsan, Republic of Korea; 2 Department of Physical and Rehabilitation Medicine, Chungnam National University Sejong Hospital, Chungnam National University College of Medicine, Sejong, Republic of Korea; Universitat de Valencia, SPAIN

## Abstract

Deafferentation and weight offloading can increase brain and spinal motor neuron excitability, respectively. End-effector gait robots (EEGRs) can blend these effects with stereotyped movement-induced neuroplasticity. The authors aimed to evaluate the usefulness of EEGRs as a postoperative neuro-muscular rehabilitation tool. This prospective randomized controlled trial included patients who had undergone unilateral total knee arthroplasty (TKA). Patients were randomly allocated into two groups: one using a 200-step rehabilitation program in an EEGR or the other using a walker on a floor (WF) three times a day for five weekdays. The two groups were compared by electrophysiological and biomechanical methods. Since there were no more enrollments due to funding issues, interim analysis was performed. Twelve patients were assigned to the EEGR group and eight patients were assigned to the WF group. Although the muscle volume of the quadriceps and hamstring did not differ between the two groups, the normalized peak torque of the operated knee flexors (11.28 ± 16.04 Nm/kg) was improved in the EEGR group compared to that of the operated knee flexors in the WF group (4.25 ± 14.26 Nm/kg) (*p* = 0.04). The normalized compound motor action potentials of the vastus medialis (VM) and biceps femoris (BF) were improved in the EEGR group (*p* < 0.05). However, the normalized real-time peak amplitude and total, mean area under the curve of VM were decreased during rehabilitation in the EEGR group (*p* < 0.05). No significant differences were found between operated and non-operated knees in the EEGR group. Five-day EEGR-assisted rehabilitation induced strengthening in the knee flexors and the muscular reactivation of the BF and VM after TKA, while reducing the real-time use of the VM. This observation may suggest the feasibility of this technique: EEGR modulated the neuronal system of the patients rather than training their muscles. However, because the study was underpowered, all of the findings should be interpreted with the utmost caution.

## Introduction

Several types of gait robots have been investigated for use in stroke patients and patients with spinal cord injuries [1, 2]. These robots can be classified as either involving therapeutic robotics, in which interactive robots facilitate recovery, or neurorobotics, in which patients are in a harness utilizing neuron connectivity [3]. In the patients with strokes and spinal cord injuries, repeated kinematic movements of hemiparetic limbs induce cortical plasticity in the topographically corresponding motor neurons, regardless of whether movements are passive or active [4, 5]. This process is called stereotyped movement-induced brain plasticity [6]. Correspondingly, rehabilitation robots are able to take advantage of this plasticity to drive changes in the nervous system of the stroke and spinal cord injured patients [7, 8].

Similarly, cortical plasticity for movement in a specific body part can also be induced in those without central nervous system injuries. The cortical plasticity of kinematic movements was observed in 20 healthy individuals shortly after repetitive thumb exercises for 5–30 minutes [4], and the cortical plasticity of kinematic movements of the elbow was observed in 22 healthy volunteers after stereotyped training for 10 minutes [5]. This rapid neuroplasticity of kinematic locomotion can also be provoked by a 10-minute robot-assisted training, and the effects were found to persist for up to 2 hours in 39 healthy adults [7]. Gait robots have shown consistency and repeatability, making them predictable neuromodulating tools that can rapidly induce stereotyped movement-induced brain plasticity in healthy individuals.

The idea that deafferentation showed increased peripheral motor neuron excitability was first introduced in an animal experiment in 1956 [9]. The definition of deafferentation represents wide ranges of neuropathophysiological conditions involving the afferent disconnection of the motor system from the central nervous system or from the peripheral sensory systems [10, 11]. That is, deafferentation describes an increase in cortical motor neuron excitation in the brain by the muscle only without any input from the central nervous system or the peripheral sensory system. Initially, this effect was induced directly by the afferent interruption of the peripheral sensory nerves. However, the methods have evolved and now focus on the indirect dissociation of the afferent sensory systems from the motor systems using local anesthesia or ischemic nerve compression [12, 13].

Furthermore, bearing weight on the legs can affect α-motor neuron excitability in the spinal cord during ambulation, as the activation of motor neuron excitability depends on the amount of weight the legs are bearing [14–17]. That is, bearing more weight induces less motor neuron excitation in the spinal cord. The offloading of body weight using gait robots can take advantage of the activation of spinal motor neurons.

In contrast to exo-skeleton type robots (EXSRs), which only control weight bearing, end-effector gait robots (EEGRs) can provide deafferentation using an end effector with a guidance force of 100% in addition to weight bearing control [18]. Therefore, EEGRs may take more advantage of brain and spinal plasticity than EXSRs. A pilot trial in 10 healthy volunteers with an EEGR that could control both deafferentation and weight bearing showed that the knee flexors and extensors could be strengthened, while reducing the real-time use of the same muscles [19]. Nevertheless, no reports on the neuroelectrophysiological role of EEGRs in knee function rehabilitation are yet available.

Changes in neuromuscular control before and after total knee arthroplasty (TKA) have been reported on a local/muscle level as well as on larger levels that attempt to describe the overall control strategy for patients undergoing TKA. Many patients who cannot walk after TKA have difficulty bearing their full body weight on their legs due to pain, poor muscular activation, or muscle atrophy [20–22]; it can take more than 2 years to fully regain preoperative levels of quadriceps femoris muscle activation, one of the most important prognostic factors after TKA [22]. Furthermore, increased knee stiffness might lead to altered biomechanics in the operated leg compared with the non-operated leg during walking after unilateral TKA [23]. Moreover, knee pain is expected to increase, regardless of the presence of obesity, in individuals over 60 years of age according to the National Health and Nutrition Examination Survey and Framingham osteoarthritis trial [24]. Although obesity, which is assessed by waist circumference, has been shown to hinder the gait speed of and distance covered by patients with knee osteoarthritis during walking in Osteoarthritis Initiative trials [25], the improvements in the frontal and sagittal tibiofemoral kinematics during gait that were induced by a reduction in body weight were not related to knee pain improvement in obese patients (body mass index of more than 35) [26]. Considering the aforementioned findings, regaining their preoperative levels of functional activity is usually delayed, especially in elderly individuals.

In contrast to the negative effects related to prognoses after TKA, Berth *et al*. reported that maximal voluntary activation of the quadriceps femoris muscles spontaneously and partially recovered within 25 months after TKA [27]. The authors stressed the importance of immediate rehabilitation after TKA to reduce deficits in the voluntary activation of the quadriceps femoris muscle. Moreover, this improvement in impaired quadriceps femoris muscle activation can occur within approximately 1 to 6 months after TKA with neuromuscular electrical stimulation [21, 28]. Additionally, preoperative neuromuscular electrical stimulation may be useful in combination with or instead of postoperative stimulation [29, 30]. However, it takes several months, at minimum, for the deteriorated neuromuscular control to reach the preoperative level. If impaired neuromuscular control, especially voluntary muscle activation, after TKA can be recovered earlier than expected, patients may experience better results in the subsequent steps of TKA recovery (strengthening programs). In addition, the central nervous system of patients undergoing TKA is not damaged, apart from the impairment due to knee disease; thus, their central nervous system is ready for neuroplastic stimuli. The efficiency of voluntary muscular activation is primarily controlled by the corticospinal tract [31]. Another benefit is that peripheral sensory awareness, especially single limb balance, can spontaneously improve quickly (around 11 days) after TKA, and this improvement has been shown to occur in patients who had poor balance before TKA [20]. Furthermore, gait robots might improve knee joint sense in patients undergoing TKA [32]. These findings may make these patients particularly suitable for interventions with an EEGR in the early postoperative period. Therefore, authors hypothesized that EEGRs for TKA patients can take advantage of neuroplasticity provoked by deafferentation, weight bearing reduction, and stereotyped movement such that this provoked neuroplasticity can rapidly restore muscular strength, reverse atrophy, and induce reactivation [33–35]. To test this hypothesis, authors compared computed tomography (CT) results and biomechanical and electrophysiological parameters at the muscular and supramuscular corticospinal level in patients undergoing ambulation rehabilitation by using an EEGR or by walking on a floor. Additionally, authors evaluated whether undergoing unilateral TKA (local factors) could affect the systemic development of this type of neuroplasticity in both legs.

## Materials and methods

This prospective, randomized, single-blind controlled trial was performed at a tertiary medical center/university teaching hospital from November 2016 to May 2017. The study protocol was approved by the Ulsan University Hospital Institutional Review Board (UUH 2016-08-036) and was registered at the Protocol Registration and Results System (PRS), www.clinicaltrials.gov, (NCT02962453: https://clinicaltrials.gov/ct2/show/NCT02962453?term=NCT02962453&rank=1). The study was conducted according to the Declaration of Helsinki. The individual in this manuscript has given written informed consent (as outlined in PLOS consent form) to publish these case details. To evaluate whether neuroplastic changes might be different between the operated and non-operated knees, the study included patients who had undergone unilateral TKA. Patients with central nervous system diseases, peripheral neuropathies, myopathies, or local muscle injuries around their thighs; patients who presented with any ambulatory impairments, decreased consciousness (Mini-Mental State Examination score < 23), or any unstable leg joints; patients with a history of arthroplasty of the legs or cardiac pacemaker implantation; and patients who refused to participate were excluded.

The enrolled patients were randomly allocated 1:1 to two groups in block-randomized order with a block size of 6, as determined by a random assignment generator (Wichmann–Hill random number generator^®^), with assignments delivered to one physical therapist in a sealed envelope. Instead of comparing patients with a sham group (only robot exposure with full weight bearing and without deafferentation), EEGR-assisted rehabilitation was compared with the gold standard of rehabilitation after TKA (ambulation training on a floor as early as possible). One group was subjected to 200-step rehabilitation using the EEGR (Morning Walk^®^, Curexo, Republic of Korea), with a step length of 40 cm, a cadence of 40 cycles/minute, a speed of 0.98 km/hour, a guidance force of 100%, and a body weight support of 100%. The gait parameters of step length, cadence, and speed were selected after considering the postoperative state. The degree of guidance force and body weight support was chosen to maximize the effect of deafferentation- and weight offloading-induced neuroplasticity, as referred to in previous reports [16, 18]. The second group was subjected to 200-step rehabilitation using walkers on a floor (WF); this treatment was conducted at a comfortable pace because the same speed as that of the 200-step rehabilitation protocol could not be guaranteed following TKA. Rehabilitation was performed three times per day for five consecutive weekdays. Measured data from the two groups were compared with respect to time (before, 3 days and 5 days after the intervention). All patients were provided identical physiotherapy, consisting of infrared radiation at a wavelength of 770–1500 *n*m, 45 cm from the patient’s body, and perpendicular to the body surface, and continuous passive range of motion (ROM) exercises for 20 minutes.

The EEGR was equipped with a surrounding safety bar for manual grasping, a frontward chest support with a Velcro^®^ strap to secure the trunk, a saddle for pelvic weight bearing, and a pair of end-effectors. The upper surface of the end-effectors completely contacted the patient’s soles in the upright position and was persistently secured with ankle, mid-foot, and forefoot Velcro^®^ straps, but free movement of the forefeet, ankles, and knees was allowed. An articulated robot arm with 3-degrees-of-freedom (DOF) was located at each foot, and the robot arm had the footplate shape end-effector. The gait trajectory of the patient’s feet was provided by the footplate. The patient’s lower limb motion was guided along the foot movement trajectory. The knee motion of walking could be made through the motion of the foot because the knee joint was constrained by the joint structure, such as the bones, muscles, and ligaments. The robot had force sensors in the saddle and footplates. The sensors in the saddle could detect the upward or downward trajectory of the pelvis and adjust to its undulation to maintain the predetermined weight support and the sensors in the foot plates could detect ground reaction force on each foot in real-time during training. The amount of body weight support by the saddle and the amount of the ground reaction force on each foot plate were displayed on the monitor in real time. For example, in the case of 100% guidance, the entire weight of the patient is supported by the robot, and the whole movement of the legs is performed in full by the EEGR, the same method as that in Blicher *et al*.’s trial [18]; on the monitor, the gauge for body weight support would display 100% of patient’s weight and the ground reaction force gauges would indicate zero. For safety issues, if an excessive alteration in the symmetry between the feet or in the predetermined trajectory of the end-effectors on the ground reaction force was detected, the EEGR would be stopped instantly. A physiotherapist could also terminate its operation by pushing a button. Compared with the well-known EEGR, the Gait Trainer^®^ (Reha-stim, Germany), for which only the velocity and step length parameters can be adjusted, the Morning Walk^®^, which was used in this study, can adapt several parameters of gait such as toe-off angle, initial contact angle, step height, step length, and cadence, corresponding to the individual status of the patients [36].

Factors collected before the intervention included patients’ demographic characteristics, diabetes mellitus status, score on the Kellgren Lawrence scale [37], knee varus angle [38], number of years and number of painful years from the diagnosis of knee osteoarthritis, number of days from TKA to the initial interventions, distances from the anterior superior iliac spine to the proximal end of the patellar tendon and from the ischial tuberosity to the fibular head, visual analog scale (VAS) scores for measuring pain [39], active ROM of motion of knee flexion and extension [40], and scores on the Western Ontario and McMaster Universities Osteoarthritis Index [41].

In calculating their volumes, the thigh muscles were assumed to have bidirectional conical shapes toward both ends. The cross-sectional areas of the quadriceps femoris and biceps femoris short and long heads, and the semitendinosus and semimembranosus muscles were calculated using the manual tracking method with PiViewSTAR software (INFINITT CO. Ltd., Seoul, Republic of Korea) at the level of trisecting the entire length of the femur were measured on CT scans, and were used to reconstruct the three-dimensional volumes of the knee flexors and extensors.

In assessing cortical motor neuron excitability, the motor evoked potential of the vastus medialis and biceps femoris long head were measured electrophysiologically (Medelec Synergy^®^, Vickers Medical, England), as described previously [42, 43]. Patients were asked to lay down in the supine or prone position and were asked to maintain the resting tone of their legs at an intensity of 5% of the maximal isometric contraction. Surface active electrodes were attached to the belly montage above the corresponding motor points [44], and a ground electrode was attached on the dorsal side of the patient’s wrist. Using a 70 mm figure-eight air film coil (Magstim Company, England), with a maximum output of 2 Tesla, trans-cranial magnetic single biphasic stimulation (pulse duration, 400 μs; interpulse interval, 12 s) was manually delivered to the scalp above the hot-spot of the vastus medialis or biceps femoris long head with a handle directed backward and tilted 45 degrees from the mid-line. The baseline motor threshold was defined as the minimum intensity at which an motor evoked potential over 50 μV was evoked at least five times in response to ten consecutive stimulations [45]. Maximal and mean peak to peak motor evoked potential amplitudes of the vastus medialis and biceps femoris long head were based on eight trials with 120% baseline motor threshold at 12-second intervals and were standardized relative to the maximal peak to peak muscle response (M-wave) amplitude.

Spinal motor neuron excitability was determined by measuring the Hoffmann (H)-reflex, the most sensitive indicator of spinal α-motor neuron excitability [15], of the vastus medialis and biceps femoris long head, as described previously [42]. Patients in the supine or lateral-decubitus position were asked to flex their knees to 15 degrees. Surface active and ground electrodes were attached [44]. Transcutaneous- or 22-gauge Teflon-covered, double lumen, inclined cannula (TECA Accessories, Oxford, England)-assisted bipolar electrical stimulation with a square-wave pulse of width 1 ms with a 12-second break was applied just lateral to the femoral artery and above the femoral triangle for the femoral nerve or between a point 2 cm below the sacral hiatus and the greater trochanter for the sciatic nerve. Twenty stimulations were applied in sequence, with increases in 0.2 V increments until the H-reflex amplitude did not increase. The maximum peak to peak M-wave amplitude was recorded after the decrease in the M-wave was confirmed, despite electrical stimulation at 0.2 V increments. If challenged, the duration of the stimulation was increased as high as the patients allowed for up to 50 ms. The peak to peak H-reflex amplitude was standardized relative to the maximal peak to peak M-wave amplitude.

Isometric peak torque and the maximal rate of torque development were evaluated in the knee flexors and extensors using Biodex^®^ (Biodex Medical Systems, USA) before the start and after the completion of the five-day interventions [42]. Patients were asked to perform voluntary knee flexion and extension warm-up exercises at 25%, 50%, and 75% intensity of maximal contraction for 3 minutes. Patients then sat in a chair, and their trunk, hips, and thighs were secured with Velcro^®^ straps. One of their knees was repositioned to 60 degrees flexion using a lever arm and the ankle was fixed to the lever arm with an ankle strap. Five-second peak torque was evaluated five times with a 55 second break and the maximal score was standardized relative to body mass. The maximal rate of torque development was defined as the slope from the onset of contraction to a point of peak torque over the torque versus time curve and was standardized relative to body mass.

The amplitude of compound motor action potential and the area under the curve (AUC) of the vastus medialis and biceps femoris were recorded simultaneously with eight-channel surface electromyography (EMG) (WEMG-8^®^, LAXTHA, Republic of Korea) using a 2.46 GHz Industrial, Scientific, and Medical Band. The compound motor action potential was measured for 5 minutes during peak torque evaluation and during rehabilitation with EEGR or WF on the third day as described previously [42]. Each value was selected from the moving averages of the smoothed signals for over 1 second and were standardized relative to the baseline amplitude or area of the compound motor action potential. Surface electrodes were attached to the skin in the same manner as for measurements of cortical motor excitability [44].

Any side effects, including palpations, false steps, dizziness, pain, and falling down, occurred during or 5 minutes after the interventions were recorded.

### Participants

All subjects were females, with a mean age of 71 years and a mean body weight of 61 kilograms. The demographic characteristics of the two groups were similar (Table 1).

**Table 1:** Demographic characteristics of patient groups.

		WF (n = 5)	EEGR (n = 9)	*p*-value
Age (year)^†^		74.60 ± 6.39	69.33 ± 4.74	0.19
Weight (kilogram)^†^		60.58 ± 13.46	61.52 ± 9.21	0.87
Height (centimeter)^†^		152.00 ± 3.46	156.41 ± 6.01	0.16
Years since arthritis diagnosis^‡^		4.40 ± 8.74	7.72 ± 2.09	0.89
Painful years since diagnosis^‡^		4.40 ± 11.61	7.33 ± 7.35	0.25
Day from surgery to the first intervention^†^		8.00 ± 4.06	7.22 ± 1.64	0.70
Varus angle, operated knee (degree)^†^		15.00 ± 1.00	14.67 ± 17.3	0.70
Varus angle, non-operated knee (degree)^†^		2.40 ± 1.14	2.11 ± 1.27	0.68
Length from ASIS to patellar tendon (centimeter)^†^		405.63 ± 12.28	411.55 ± 29.88	0.68
Length from ischial tuberosity to fibular head (centimeter)^†^		402.54 ± 11.83	408.13 ± 25.71	0.79
VAS (0–100)^‡^		45.80 ± 11.21	39.89 ± 11.69	0.37
Active ROM, operated knee ^‡^	Extension (-360–0)	-13.00 ± 4.47	-8.33 ± 3.54	0.06
	Flexion (0–360)	113.00 ± 6.71	116.6 7 ± 5.00	0.26
WOMAC (0–96)^‡^		71.00 ± 8.54	61.11 ± 9.71	0.08
Peak torque of knee extensor/body weight: mean of both (Nm/kg)		108.84 ± 46.04	116.72 ± 39.41	0.26
Peak torque of knee flexor/body weight: mean of both (Nm/kg)		61.91 ± 21.47	69.04 ± 26.48	0.14
Sex: male/female (%)^§^		0 (0.0) / 5 (100.0)	3 (33.3) / 6 (66.7)	0.25
DM: absence/presence (%)^§^		3 (60.0) / 2 (40.0)	8 (88.9) / 1 (11.1)	0.50
Operated side: left/right (%)^§^		4 (80.0) / 1 (20.0)	6 (66.7) / 3 (33.3)	1.00
KLS, operated knee (%)^§^	I (None)	0 (0.0)	0 (0.0)	0.58
	II (Doubtful)	0 (0.0)	0 (0.0)	
	III (Minimal)	0 (0.0)	0 (0.0)	
	IV (Moderate)	4 (80.0)	5 (55.6)	
	V (Severe)	1 (20.0)	4 (44.4)	
KLS, non-operated knee (%)^§^	I (None)	0 (0.0)	0 (0.0)	1.00
	II (Doubtful)	4 (80.0)	6 (66.7)	
	III (Minimal)	1 (20.0)	3 (33.3)	
	IV (Moderate) and V (Severe)	0 (0.0)	0 (0.0)	

WF, walkers on a floor; EEGR, end-effector gait robot; ASIS, anterior superior iliac spine; VAS, visual analog scale; ROM, range of motion; WOMAC, Western Ontario and McMaster Universities Osteoarthritis Index; DM, diabetes mellitus; KLS, Kellgren Lawrence scales.

^†^ Independent t-test,

^‡^ Mann-Whitney U test,

^§^ Fisher’s exact test.

### Statistics

Based on a previous report [19], the minimum mean significant difference in the peak torque of knee extensors was defined as 14.79 Nm and the standard deviation as 23.52 Nm, with a calculated standardized difference of 0.63. The minimum number of subjects per group required to yield a two-sided α of 0.05 and a power of 80% was 27, according to Altman’s nomogram [46]; allowing for a drop-out rate of 10%, a minimum of 30 patients per group were required. All two-sided statistical analyses were performed using the Statistical Package for the Social Sciences 24 (SPSS Inc., USA). Normal distribution, skewness, and kurtosis were assessed using the Shapiro-Wilk or Kolmogorov-Smirnov test, and variables not normally distributed were log transformed. The parameters of the operated knee in the WF group and those of the operated knee in the EEGR group were compared by two-way repeated measures analysis of variance (before, 3 days after and 5 days after the intervention) within and between the subjects. Changes in the parameters of the operated and non-operated knees in the EEGR group from before the intervention to 5 days after the intervention were assessed by paired measures analysis (paired T-test). On the third day after the intervention, Mann-Whitney U tests were conducted twice to compare the operated knee in the WF group with the operated and non-operated knees in the EEGR group. Demographic data were analyzed by two sample independent t-tests (age, weight, height, day, varus angle, length), Mann-Whitney U tests (year, VAS, ROM, Western Ontario and McMaster Universities Osteoarthritis Index), or Fisher’s exact tests (sex, diabetes mellitus, operated side, Kellgren Lawrence scale), as appropriate.

## Results

**Fig 1:**
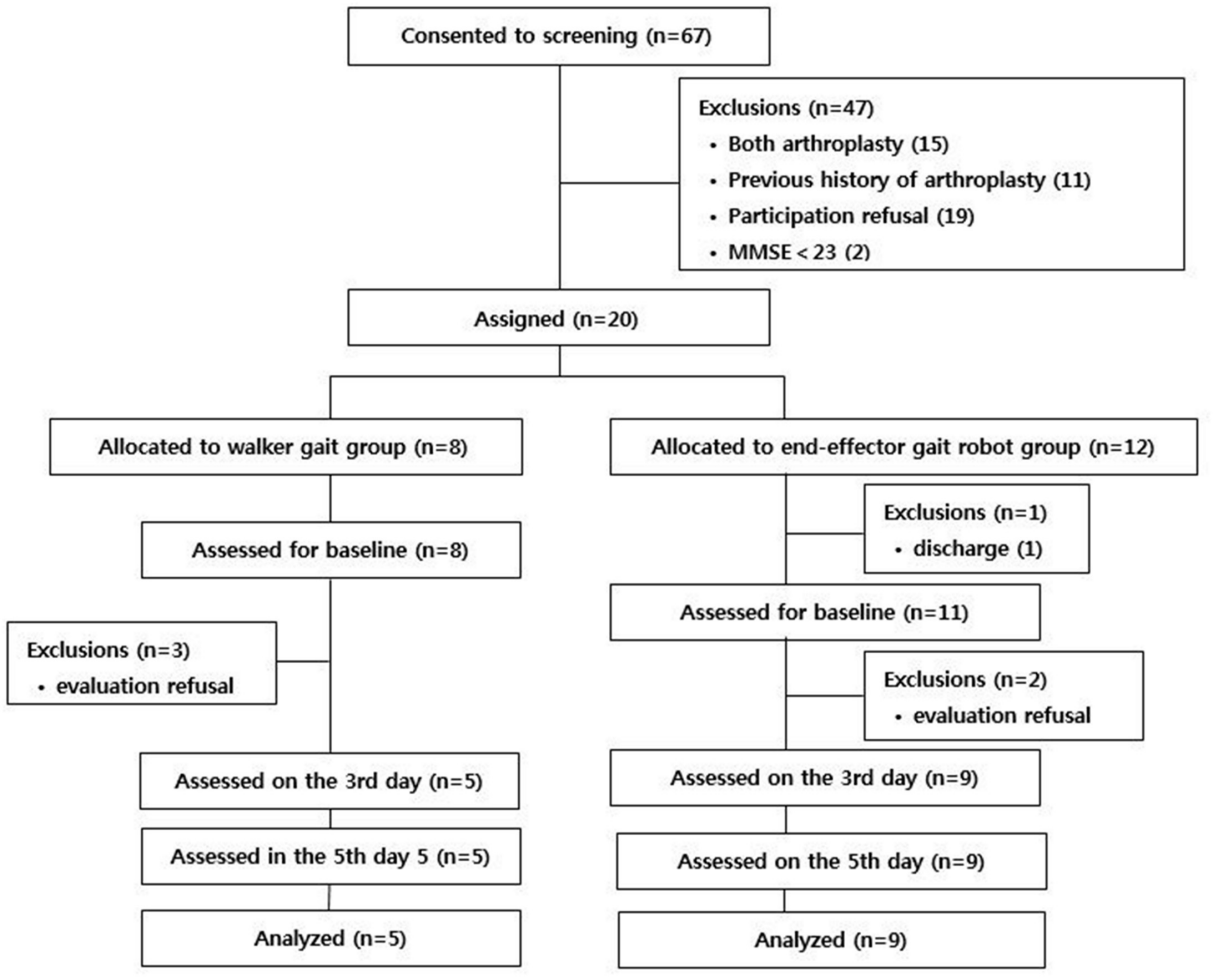
The flow diagram. (MMSE; Mini-Mental State Examination).

**Fig 2:**
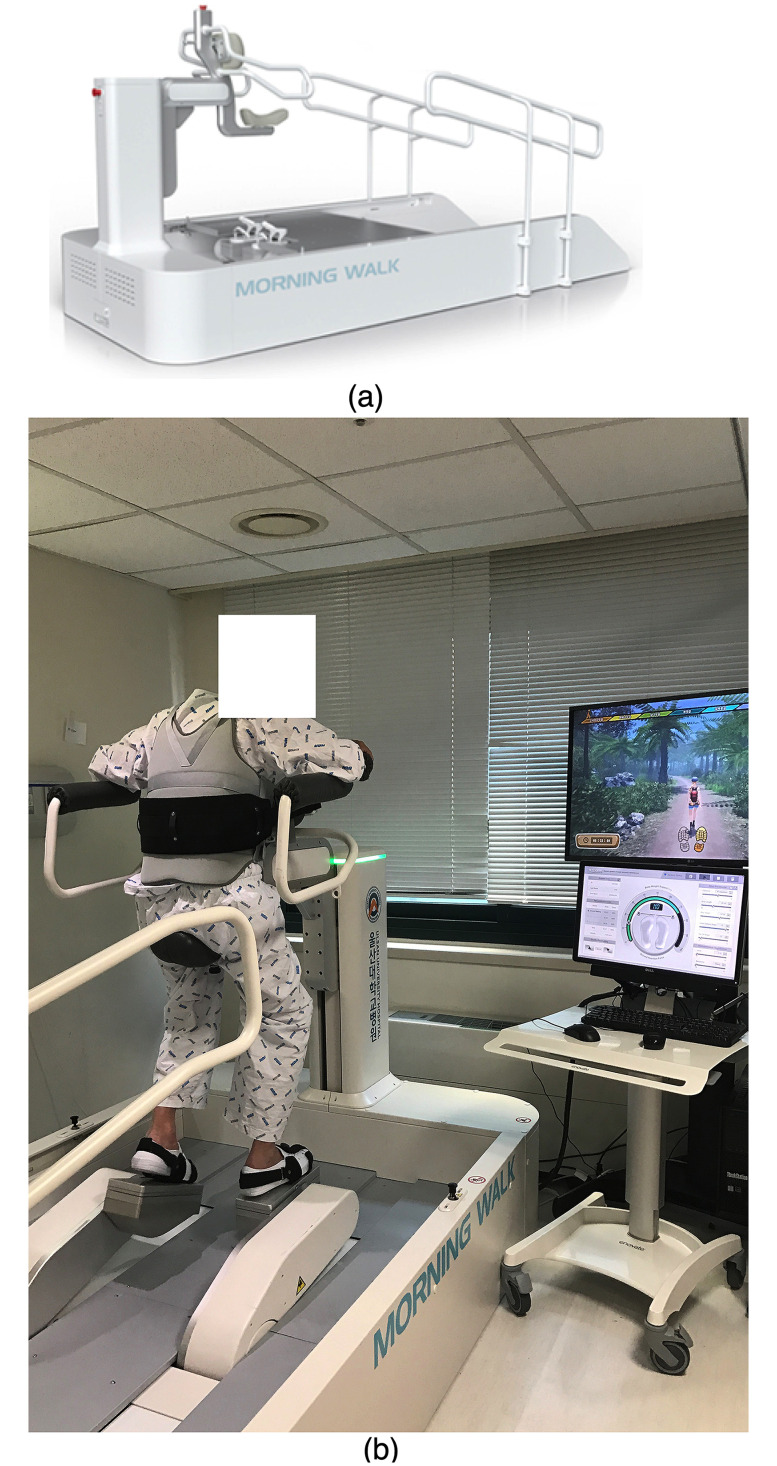
(A) The conceptual image of the end-effector gait robot. (B) The patient is doing the ambulation rehabilitation on the saddle of the end-effector gait robot.

Due to unexpectedly slow enrollment, the authors requested an extension of the trial period to the National Research Foundation; however, no further funding was permitted; thus, interim analysis was performed. Because in-patient management after TKA was fully provided until discharge by the national medical insurance system, all the screened patients were in-patients. Of the 67 patients screened, 47 were excluded: 15 due to bilateral TKA, 11 due to previous arthroplasty, 19 due to their own refusal, and 2 due to a Mini-Mental State Examination score under 23. Of the 20 patients enrolled, 12 were assigned to the EEGR group and 8 to the WF group. Among the 20 patients enrolled, 6 patients dropped out: 1 due to his/her discharge and 5 due to their own refusal to undergo follow-up assessment. Ultimately, 9 patients in the EEGR group and 5 patients in the WF group were analyzed (Figs 1 and 2).

Regarding the responsiveness of H-reflexes in the vastus medialis and biceps femoris, all of the patients showed clear evoked responses in no more than one third of the 20 stimulations.

### Cross-sectional areas and volumes of knee flexors and extensors

The cross-sectional areas of the quadriceps femoris and of the hamstring muscles did not differ among the operated knees in the WF group and the operated and non-operated knees in the EEGR group on three levels. The reconstructed three-dimensional volumes of the knee flexors and extensors in these three groups did not differ significantly (S1 Table).

### Peak torque, compound motor action potential, and maximal rate of torque development

**Fig 3:**
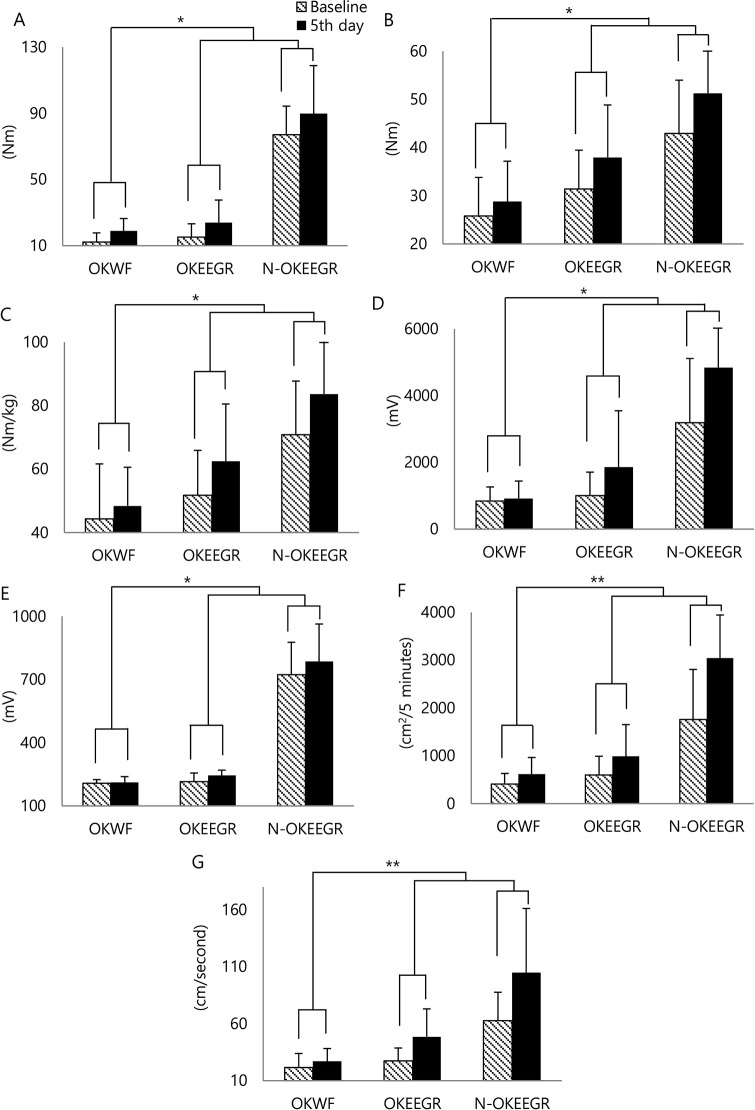
The peak torque and electromyographic muscle activation of knee flexors and extensors. Compared with the operated knees in the WF group (OKWF), the operated knees in the EEGR group (OKEEGR) and the non-operated knees in the EEGR group (N-OKEEGR) showed more improvement in (A) the peak torque of the knee extensors, (B) the peak torque of the knee flexors, (C) the peak torque of knee flexors/body weight, (D) the peak amplitude of the vastus medialis, (E) the mean amplitude of the biceps femoris, (F) the total area of the vastus medialis, and (G) the mean area of the vastus medialis. *p < 0.05, **p < 0.01 by two-way repeated measures analysis of variance or paired T-test.

Following the intervention, the peak torque improved in the operated knees of the WF group and in the operated knees of the EEGR group. The normalized peak torque of the knee flexors in the operated knees, standardized by patient body weight, significantly improved over time to a greater extent within the subjects in the EEGR group (11.28 ± 16.04 Nm/kg: 51.82 ± 14.08 to 62.50 ± 18.01) than within those in the WF group (4.25 ± 14.26 Nm/kg: 44.27 ± 17.37 to 48.42 ± 12.15) (*p* = 0.04, F: 11.64). In addition, the nonnormalized peak torque in the extensors of the operated knees significantly improved over time within the subjects; it was at least 1.3 times higher in the EEGR group (9.11 ± 10.35 Nm: 15.23 ± 8.04 to 24.02 ± 13.56) than in the WF group (6.88 ± 6.55 Nm: 12.10 ± 5.66 to 18.98 ± 7.44) (*p* = 0.04, F: 9.52). Similarly, the nonnormalized peak torque in the flexors of the operated knees significantly improved over time within the subjects and was at least 2.1-fold higher in the EEGR group (6.60 ± 9.41 Nm: 31.37 ± 8.09 to 37.97 ± 10.87) than in the WF group (3.03 ± 7.66 Nm: 25.84 ± 7.97 to 28.81 ± 8.36) (*p* = 0.03, F: 13.56). Compared with Cho *et al*.’s report, in which female patients with a similar mean age (61.7 years) showed 62.43 Nm (mean value) of peak torque in the knee extensor before TKA [20], the current patients manifested no more than a 60% reduction in peak torque following TKA such that 2.23 Nm (approximately 10% of the postoperational values) of peak torque change in the knee extensor might be clinically relevant. Meanwhile, the WF group showed profound weakness in the knee flexors and extensors before the intervention according to the starting torques in the EEGR and WF groups. While there were significant differences between the two groups, no significant differences were observed between the operated and non-operated knees in the EEGR group: the normalized peak torque of the knee flexors standardized by patient body weight (11.28 ± 16.04 Nm/kg versus 13.11 ± 20.86 Nm/kg); the nonnormalized peak torque of the knee extensors (9.11 ± 10.35 Nm versus 12.64 ± 23.17 Nm); the nonnormalized peak torque of the flexors (6.60 ± 9.41 Nm versus 9.73 ± 13.20 Nm) (Fig 3 and S2 Table).

Although most compound motor action potentials in two groups improved after intervention, the operated knees in the EEGR group showed larger improvements in time within the subject than those in the operated knees in the WF group, with respect to the normalized peak amplitudes of compound motor action potential (861.15 ± 1200.33 mV versus 83.49 ± 472.33 mV), normalized total areas (391.43 ± 529.44 cm^2^/5 minutes versus 204.55 ± 283.66 cm^2^/5 minutes), and normalized mean areas of AUC in the vastus medialis (21.33 ± 17.84 cm^2^/second versus 6.52 ± 11.87 cm^2^/second) and the normalized mean amplitudes (30.21 ± 33.28 mV versus 4.37 ± 22.58 mV) in the biceps femoris (*p* < 0.05) (Fig 3 and S2 Table). Meanwhile, the WF group showed relatively low baseline values for most of the parameters before the intervention, as did the WF groups regarding the peak torque. Similar to the findings for peak torque, no significant differences were found between the operated and non-operated knees in the EEGR group (Fig 3 and S2 Table).

No significant differences in the interval changes in the maximal rate of torque development in knee flexors and extensors were observed among the three groups (S2 Table).

### Cortical and spinal motor neuron excitability

**Fig 4:**
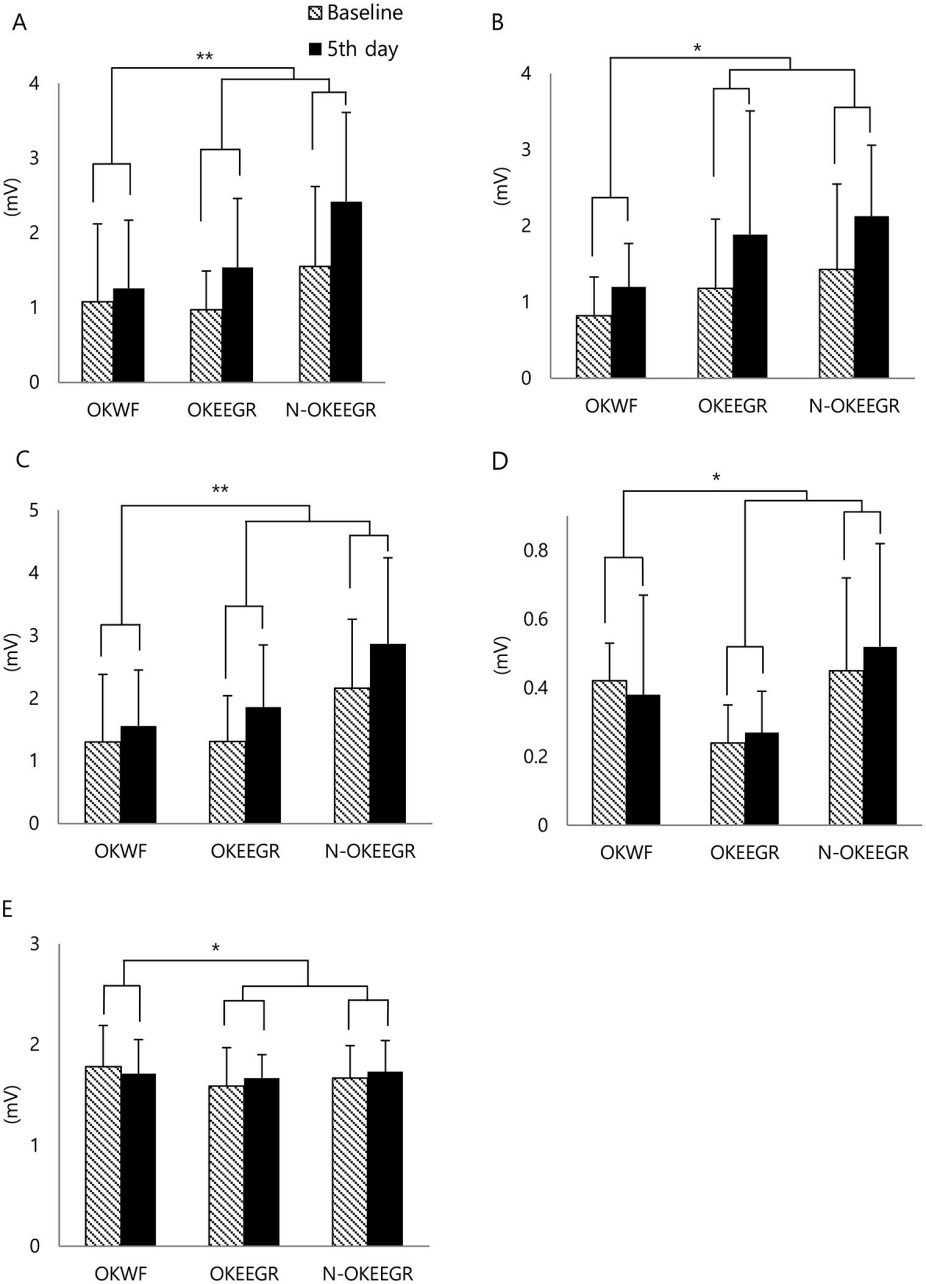
The cortical and spinal motor neuron excitability of the knee flexors and extensors. Compared with the operated knees in the WF group (OKWF), the operated knees in the EEGR group (OKEEGR) and the non-operated knees in the EEGR group (N-OKEEGR) showed more improvement in (A) the mean motor evoked potential amplitude of the vastus medialis, (B) the mean motor evoked potential amplitude of the biceps femoris long head, (C) the maximal motor evoked potential amplitude of the vastus medialis, (D) the maximal Hoffmann-reflex amplitude of the vastus medialis, and (E) the maximal Hoffmann-reflex amplitude of the biceps femoris long head. *p < 0.05, **p < 0.01 by two-way repeated measures analysis of variance or paired T-test.

The mean motor evoked potential amplitude in the vastus medialis was significantly improved in time within the subject at least 3.1 times higher in the operated knees in the EEGR group (0.57 ± 0.72 mV) than in the operated knees in the WF group (0.18 ± 0.98 mV) (*p* = 0.01, F: 6.95). Similar findings were observed for interval changes in maximal motor evoked potential amplitude (0.55 ± 0.89 mV versus 0.26 ± 0.98 mV) (*p* = 0.01, F: 18.48). Similarly, motor evoked potential amplitude in the biceps femoris was significantly improved in time within the subject at least 1.8 times higher in the operated knees of the EEGR group (0.71 ± 1.26 mV) than in the operated knees in the WF group (0.38 ± 0.54 mV) (*p* = 0.04, F: 8.34). However, none of these differences were significant after standardization relative to the maximal M-wave. Moreover, there were no significant differences between the operated and the non-operated knees in the EEGR group (Fig 4 and S3 Table): the mean motor evoked potential amplitude of the vastus medialis (0.57 ± 0.72 mV versus 0.87 ± 1.14 mV); the maximal motor evoked potential amplitude of the vastus medialis (0.55 ± 0.89 mV versus 0.77 ± 1.23 mV); the motor evoked potential amplitude of the biceps femoris (0.71 ± 1.26 mV versus 0.70 ± 1.02 mV) (Fig 4 and S3 Table).

In contrast to the decrease in the vastus medialis maximal H-reflex amplitude observed in the operated knees of the WF group (-0.04 ± 0.20 mV), this parameter was significantly improved in time within the subject in the operated knees of the EEGR group (0.03± 0.11 mV) (*p* = 0.04, F: 7.81). Similar results were observed in the maximal biceps femoris H-reflex amplitude in the operated knees in the WF group (-0.07 ± 0.37 mV) and in the operated knees of the EEGR group (0.08 ± 0.30 mV) (*p* = 0.04, F: 4.19). However, none of these differences remained significant following standardization relative to the maximal M-wave. There were no significant differences between the operated and the non-operated knees in the EEGR group: the maximal H-reflex amplitude of the vastus medialis (0.03± 0.11 mV versus 0.07 ± 0.28 mV); the maximal H-reflex amplitude of the biceps femoris (0.08 ± 0.30 mV versus 0.06 ± 0.31 mV) (Fig 4 and S3 Table).

### Real-time use of the knee flexors and extensors

**Fig 5:**
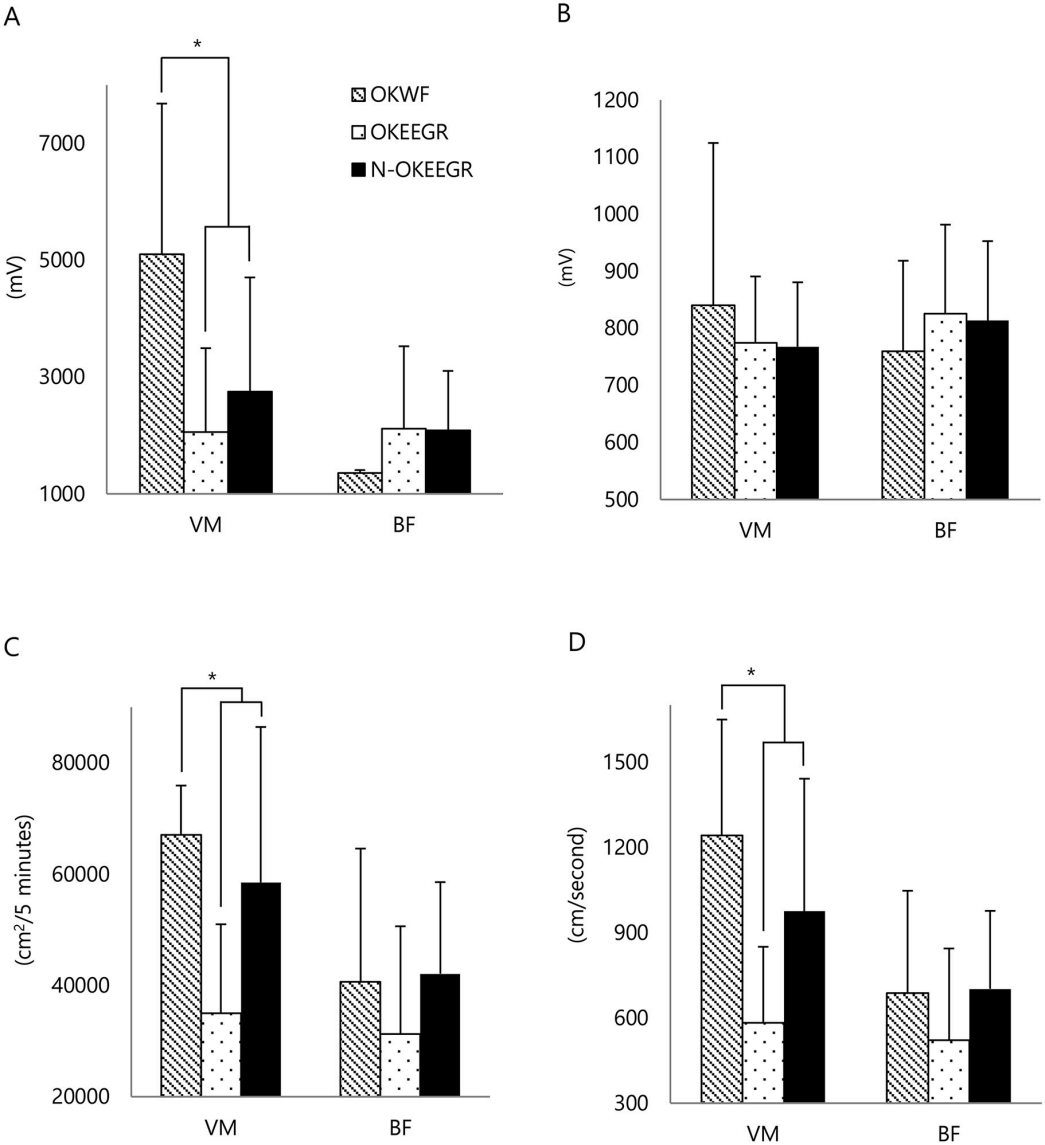
Real-time use of the knee flexor and extensor muscles. Compared with the operated knees in the WF group (OKWF), the operated knees in the EEGR group (OKEEGR) and the non-operated knees in the EEGR group (N-OKEEGR) showed reduced (A) peak amplitudes in the vastus medialis (VM) and biceps femoris (BF), (B) mean amplitudes in the vastus medialis and biceps femoris, (C) total AUCs in the vastus medialis and biceps femoris, and (D) mean area under curves in the vastus medialis and biceps femoris. *p < 0.05 by Mann-Whitney U tests or paired T-test.

During ambulation rehabilitation, the simultaneously recorded normalized vastus medialis peak compound motor action potential amplitude and the normalized vastus medialis total and mean area under the curve of the operated knees in the EEGR group (2059.44 ± 1435.54 mV, 35008.25 ± 15984.85 cm^2^/[5 minutes], 583.58 ± 266.47 cm^2^/second, respectively) were significantly smaller than those (5100.19 ± 2583.15 mV, 67090.05 ± 8836.63 cm^2^/[5 minutes], 1241.74 ± 407.56 cm^2^/second, respectively) of the operated knees in the WF group (*p* < 0.05). Similar results were observed for the simultaneously recorded normalized vastus medialis peak compound motor action potential amplitude and the normalized vastus medialis total and mean area under the curve of the non-operated knees in the EEGR group (2751.98 ± 1954.38 mV, 58511.35 ± 27945.37 cm^2^/[5 minutes], 975.38 ± 465.85 cm^2^/second, respectively) compared to those of the operated knees in the WF group (*p* < 0.05). However, there were no significant differences between the operated and the non-operated knees in the EEGR group. Meanwhile, in the biceps femoris, these variables did not differ significantly among the three groups (Fig 5 and S4 Table).

### Adverse effect

No adverse events, including palpations, false steps, dizziness, pain, or falling down, occurred during or 5 minutes after the interventions.

## Discussion

The authors hypothesized that EEGRs could cause deafferentation and weight offloading, taking advantage of brain and/or spinal neuroplasticity to drive changes in the central nervous system following TKA and thereby leading to the rapid restoration of strength, reversal of atrophy and activation of the muscles around the knee joint. This advantage was expected to lead to more efficient rehabilitation. We therefore conducted a prospective randomized trial comparing a five-day EEGR intervention effect with a five-day WF intervention effect in patients who underwent unilateral TKA. Due to funding issues, closure of the study occurred at the halfway point. However, the interim analysis yielded several key findings: (1) the normalized peak torque of the flexors and the normalized EMG activities of the vastus medialis and biceps femoris were significantly higher in the EEGR group than in the WF group; (2) these improvements in the EEGR group were not accompanied by increases in the maximal ratio of muscular recruitment or in cross-sectional areas / volumes of the same muscles; (3) the nonnormalized motor evoked potential amplitudes of the vastus medialis and biceps femoris were significantly increased by EEGR; (4) the nonnormalized H-reflex amplitudes of the vastus medialis and biceps femoris was significantly increased in the EEGR group but were reduced in the WF group; (5) the real-time normalized EMG activities of the vastus medialis were decreased in the EEGR group; and (6) these changes were not observed between the operated and non-operated knee in the EEGR group. However, recruitment stopped due to a lack of funding, and therefore, all of the findings should be interpreted with the utmost caution. Due to under-enrollment, it would not be possible to determine whether a non-significant result was from a lack of sufficient enrollment or a scientific discovery into an underlying principle. Although the current trial might have been overpowered in the beginning due to the deviated standardized difference that was calculated using the high knee extensor peak torque value in young male volunteers [19], it is unclear which differences between values could be observed with 80% power.

These findings showed that EEGR-assisted rehabilitation for 5 days significantly affected the peak torque and EMG activities of the flexors and extensors of both the operated and the non-operated knees. These results are in agreement with findings in 10 healthy volunteers, in that EEGR-assisted training for 5 minutes with deafferentation and weight offloading resulted in prompt peak torque improvement in the knee flexors and extensors [19]. Although patients in this investigation underwent unilateral TKA, improvements also occurred in the non-operated knees, with no differences occurring between the knees. These findings suggest that systemic factors, not local factors such as TKA, may be involved in these improvements.

In this study, interval changes in the cross-sectional areas were assessed at three levels to reconstruct the three- dimensional volumes of the operated and the non-operated knee flexors (quadriceps femoris) and extensors (biceps femoris, semitendinosus and semimembranosus muscles), as described in the Materials and Methods section. Although peak torque and EMG activities are dependent on muscle volume, the improved peak torque and voluntary muscular reactivation in the EEGR group were not accompanied by increases in area or volume. These findings indicate that factors at the supra-muscular level, not at the muscular level, may be involved in these improvements. However, it should be concerned that muscle volume and muscle strength are often nonlinearly related. Based on the finding that the cross-sectional area at the mid-thigh level is strongly related to the total volume of skeletal muscle in the body [47], the authors roughly estimated the volumes of the quadriceps femoris and biceps femoris short and long heads, and the semitendinosus and semimembranosus muscles at the level of trisecting the entire length of the femur on CT scans; the authors presumed that the majority of patients (more than two thirds) were suspected of having sarcopenia. However, no cut-off value have been defined for sarcopenia using CT scans [48]. Moreover, neither dual energy X-ray absorptiometry (DEXA) nor grip strength tests were performed for sarcopenia diagnosis as well [48]. Thus, the authors cannot definitely sure whether any of the participants seemed to have sarcopenia in the current population including older adults.

Brain plasticity can be rapidly induced by stereotyped movements [4, 5]. EEGRs have no limitations in terms of DOFs; thus, this technique allows two types of 200 step rehabilitations using the same stereotyped movement in each patient, which is one of the significant advantages of EEGR over EXSR. Apart from stereotyped movement-induced brain plasticity, other supra-muscular actions might occur.

As we mentioned above, EEGRs may take advantage of inducing deafferentation-induced brain plasticity [18, 49] and weight bearing offloading-induced spinal neuroplasticity [17, 18]. In the nonnormalized value, the cortical and spinal motor neuron excitability of the vastus medialis and biceps femoris were significantly increased in the EEGR group. One-third of central motor activation is due to afferent input from the muscles [50], and altering this feedback affects cortical motor excitability [51]. Similar to the rapid inhibition of GABAergic neurons in stereotyped movement-induced brain plasticity [52, 53], deafferentation by ischemic nerve blocking can rapidly reduce the GABA level by 50% [54]. Additionally, the motor evoked potential of the biceps brachii was increased up to 2.5-fold shortly after ballistic exercises following ischemic nerve blocking [12]. Similar results were also observed with end-effector gait robots; a 1.5-fold reduction in short interval intracortical inhibition was found after 20-minute passive training with 100% guidance force in 13 healthy volunteers [18]. In the case of 100% guidance force, passive leg movements by end-effectors lead to non-self-directed action [18] so that the perception of concurrent afferent input differed from that of voluntarily initiated action, leading to production of condition similar to deafferentation [55]. Moreover, the spectroscopic activation of the motor-sensory cortex after 60-second robot training with 100% guidance force in 14 healthy adults was higher than with a treadmill under the same conditions [49]. Therefore, training under EEGR-induced deafferentation may also take advantage of neuroplastic brain reactions. However, the current trial did not compare EEGRs with EXSRs, preventing quantification of the effect by deafferentation on stereotyped movement-induced brain plasticity.

In the current trial, the nonnormalized spinal motor neuron excitability of the vastus medialis and biceps femoris in both the operated and non-operated knees in the EEGR group was significantly increased, but was reduced in the operated knees of the WF group. EMG activities of the lower legs can be observed during passive ambulation, even in complete paraplegic patients [56]. The H-reflex amplitude of the soleus was reduced in healthy people by 25% immediately after bicycling [16]. Moreover, the H-reflex amplitude was inhibited in healthy people, even in the upper extremities, after 10-minute gait training [17]. Similarly, gait robot training with no reduction in weight bearing suppressed spinal motor neuron excitability, reducing the motor evoked potential amplitude of the tibialis anterior. However, training with 33% body weight reduction did not inhibit spinal excitability or motor evoked potential in healthy individuals [18]. Spinal motor neuron excitability in the current trial may have been activated by the offloading of 100% of body weight.

Methodologically, the combination of the 100% guidance force and weight offloading might have induced synergistic or additive effects on neuroplasticity. However, no significant differences were observed in the normalized values. Therefore, in the current trial, it was not easy to clarify whether EEGR contributed synergistic or additive effects to neuroplasticity. Meanwhile, the EXSR did not significantly induce neuromodulation; Beta and mu rhythms in the brain cortex were more inhibited during Lokomat^®^-assisted treadmill ambulation than during treadmill ambulation alone [57]. Moreover, self-directed engagement in Lokomat^®^-assisted training increased the muscular activation in the lower extremities more than passive engagement [58]. In the current trial, it was not confirmed whether self-directed engagement in the EEGR might occur under the condition of 100% guidance force. Future trials are needed to assess how the brain and spinal cord interact with each other in these conditions and the degree of mental activity required for patients to engage in robot-assisted training.

The maximal rate of torque development is defined as the speed of the recruitment of peripheral motor units during the force-time curve [59], so that increases in brain and/or spinal cord neuroplasticity should result in increases in the maximal rate of torque development. In our observation, no significant differences in the maximal rate of torque development were found. This observation might be due to the interim analysis with a small number of subjects. Because the current trial enrolled only adults without central nervous system diseases, their full degree of motor unit recruitment may have been reached during isometric contractions, resulting in a potential ceiling effect, with no further increases in maximal rate of torque development. Additionally, the lack of significant differences between operated and non-operated knees in the EEGR group could rule out poor recruitment due to pain following TKA. Because discordances between time intervals and maximal rate of torque development quantification were observed in healthy persons [60], assessment over the entire signal period may have influenced the results of maximal rate of torque development. Additional studies are required to determine the reasons underlying the lack of significant differences in the maximal rate of torque development.

The current trial showed that the normalized mean, total area under the curve, and the normalized peak of the compound motor action potential amplitude of the vastus medialis were significantly lower in the operated and the non-operated knees of the EEGR group than in the operated knees in the WF group, as determined by real-time monitoring of EMG activities. This result might be attributed to the fact that the deafferentation and the weight offloading of EEGR made muscles highly ready for being recruited to perform the same rehabilitation (200 steps) through the enhanced brain and spinal motor neuron excitability. However, an EEGR trial using the Haptic Walker^®^ enhanced recruitment of the vastus medialis, vastus lateralis, and biceps femoris, but the amount of body weight support was uncontrolled [61]. In contrast, EXSRs have reported the opposite EMG activities; Gait training with Lokomat^®^, a famous EXSR, increased EMG activities in the thigh muscles in healthy individuals [58, 62]. However, the applied control algorithm in these two trials was either error augmentation or assist-as-needed, and the additional torque or inertia of exoskeletons can influence muscle activities [62, 63]. The G-EO system^®^, another EXSR, yielded results contrasting those observed in this trial, including the increased activation of the vastus medialis and vastus lateralis over all gait cycles [3]. However, that trial enrolled patients with hemiparetic limbs and did not standardize the speed and amount of body weight support; moreover, physical therapists provided manual assistance to help these patients extend their knees. EMG activities of the leg muscles in EXSRs also depend on impedance, and they have a limited DOF [1, 64]. Therefore, EXSRs are suspected to activate patients’ own muscles to overcome additional torque or inertia of the exoskeletons themselves. Meanwhile, movement errors can increase motor cortex excitability [65, 66]. Unlike a pre-determined training trajectory in EXSRs, EEGRs with free DOF may enhance movement error-activated neuroplasticity, resulting in a positive effect on the enhanced motor pathway.

### Limitations

The current findings should be interpreted with caution due to the fact that it is an interim, per-protocol analysis [67] with single-blind trials, a high drop-out rate (30%), and no sham group (only robot exposure). The sample size was quite limited, indicating that this study might only be useful to determine the feasibility of the proposed technique, and it is still unclear whether the few negative findings were due to some underlying scientific principle or lack of statistical power. In a future study, the analysis of the results under a more general population should be considered. Many measurements can make the statistical analysis be susceptible to the problem of multiple comparisons, and any statistical rectifications such as Bonferonni corrections were not conducted to address this issue. Additional studies may be required to determine whether operated and non-operated knees differ in neuroplasticity and to determine the reasons underlying the lack of significant differences in standardized motor evoked potential, H-reflex, and real-time biceps femoris use.

Walking speed can affect the EMG activities of the leg muscles [68]. As ambulation speed increases in EXSRs (from 1.5 to 3.0 km/hour and from 2.7 to 6.2 km/hour), brain activation by spectroscopy and EMG activities of the legs are increased proportionally in healthy adults [49, 68]. Although no change in muscular activities was observed when walking speed was increased from 1.5 to 2.7 km/hour in healthy individuals [62], it remains unclear whether the speed used in the current study, 0.98 km/hour, was optimal.

Although the ground reaction forces in end-effectors change under various gait conditions [69], these forces were not measured. Finally, functional parameters, including VAS and Western Ontario and McMaster Universities Osteoarthritis Index scores, were collected in the current trial so that they could be compared; actually, a similar comparison was conducted in the authors’ previous trial, in which both the Western Ontario and McMaster Universities Osteoarthritis Index and VAS scores spontaenously improved (60.2 ± 6.9 to 55.1 ± 12.5 and 76.3 ± 10.9 to 48.8 ± 13.5, respectively) from 4.5 days before to 11.3 days after TKA [20]. Furthermore, the functional parameters (Berg balance scores, functional ambulation scores, 6-minute walking distances and 10-meter sitting to standing time) were significantly improved by a gait robot-assisted rehabilitation in patients undergoing TKA [32]. The literature suggests that outcomes studies following TKA should include patient-reported and performance-based outcomes. Perhaps the strength and activation data can be considered performance based, but the lack of patient-reported outcomes, such as the Western Ontario and McMaster Universities Osteoarthritis Index is a bit concerning and detracts from the overall impact of the paper.

In practical terms of healthcare costs, whether the extra cost of using the robot justifies the authors’ proposal should be taken into consideration; the price of the current EEGR (Morning Walk^®^) is approximately 300,000 USD, and one of the most popular commercialized EXSR (Lokomat^®^) costs 330,000 USD. However, traditional physiotherapy, which involved infrared radiation and continuous passive ROM exercises in the current trial, generally costs only 1,000 USD per patient for 1 month in the authors’ country.

## Conclusions

Five-day EEGR-assisted rehabilitation with deafferentation and weight offloading showed more efficient biceps femoris and vastus medialis strengthening after unilateral TKA, while reducing the real-time use of the vastus medialis. However, it did not restore muscular volume or activation. The current changes were not also affected by the preceding TKA. Despite this being an interim analysis, this investigation might suggest the feasibility of this technique; EEGR could modulate the neuronal system of patients rather than the trained muscles of the patients. Additional studies are required to determine the reasons underlying the lack of significant differences in standardized motor evoked potential, H-reflex, maximal rate of torque development, and real-time biceps femoris use. Because the study was underpowered, all of the findings should be interpreted with the utmost caution.

## Supporting information

S1 Table Cross-sectional areas and volumes of knee flexors and extensors. WF training with walkers on a floor; EEGR training with end-effector gait robot, Extensors include the quadriceps femoris; Flexors include the biceps femoris, semitendinosus and semimembranosus muscles, *p*-value by Mann-Whitney U tests or paired T-test.
(DOCX)

S2 Table The peak torque and electromyographic muscle activation of knee flexors and extensors. WF training with walkers on a floor; EEGR training with end-effector gait robot, EMG electromyography, VM vastus medialis, BF Biceps femoris; AUC area under the curve, *p*-value by two-way repeated measures analysis of variance or paired T-test.
(DOCX)

S3 Table The cortical and spinal motor neuron excitability of the knee flexors and extensors. WF training with walkers on a floor; EEGR training with end-effector gait robot; MEP motor evoked potential; BF biceps femoris; VM vastus medialis, H-reflex Hoffmann-reflex, *p*-value by two-way repeated measures analysis of variance or paired T-test.
(DOCX)

S4 Table Real-time use of the knee flexor and extensor muscles. EMG electromyography; WF training with walkers on a floor; EEGR training with end-effector gait robot; VM vastus medialis, BF Biceps femoris, CMAP compound motor action potential; AUC area under the curve, *p*-value by Mann-Whitney U tests or paired T-test.
(DOCX)

S1 Protocol The registered protocol (https://www.protocols.io/). (PDF)

S1 Checklist CONSORT 2010 checklist of information to include when reporting a randomised trial*. (DOC)
