# Supplementary material for: Five-day rehabilitation of patients undergoing total knee arthroplasty using an end-effector gait robot as a neuromodulation blending tool for deafferentation, weight offloading and stereotyped movement: Interim analysis
Source: PLoS One. 2020 Dec 16;15(12):e0241117. doi: 10.1371/journal.pone.0241117 (PMC7743990; doi:10.1371/journal.pone.0241117)
Supplement: S1 Table — WF training with walkers on a floor; EEGR training with end-effector gait robot, Extensors include the quadriceps femoris; Flexors include the biceps femoris, semitendinosus and semimembranosus muscles, p-value by Mann-Whitney U tests or paired T-test. (DOCX) [file pone.0241117.s001.docx]

|  | | ①Operated knee in WF (n=5) | ②Operated knee in EEGR (n=9) | ③Non-operated knee in EEGR (n=9) | *p*-value |
| --- | --- | --- | --- | --- | --- |
| Cross-sectional  area of proximal  1/3 extensors (cm^2^) | Baseline | 29.25 ± 12.10 | 26.03 ± 10.69 | 29.65 ± 12.26 | 0.24 (① vs. ②)  0.21 (② vs. ③) |
|  | 5^th^ day | 27.64 ± 14.28 | 27.34 ± 6.98 | 30.41 ± 8.73 |  |
| Cross-sectional  Area of proximal 1/3 flexors (cm^2^) | Baseline | 2.30 ± 1.77 | 1.55 ± 18.45 | 2.40 ± 3.60 | 0.52 (① vs. ②)  0.18 (② vs. ③) |
|  | 5^th^ day | 1.81 ± 2.03 | 1.25 ± 1.61 | 2.45 ± 1.87 |  |
| Cross-sectional  area of middle 1/3  extensors (cm^2^) | Baseline | 39.39 ± 13.17 | 41.79 ± 16.15 | 42.45 ± 9.55 | 0.95 (① vs. ②)  0.87 (② vs. ③) |
|  | 5^th^ day | 42.64 ± 10.03 | 46.66 ± 11.69 | 47.07 ± 10.86 |  |
| Cross-sectional  area of middle 1/3  flexors (cm^2^) | Baseline | 26.23 ± 12.89 | 24.10 ± 13.88 | 19.81 ± 6.25 | 0.64 (① vs. ②)  0.37 (② vs. ③) |
|  | 5^th^ day | 20.18 ± 3.90 | 21.01 ± 5.59 | 22.02 ± 6.27 |  |
| Cross-sectional  area of distal 1/3  extensors (cm^2^) | Baseline | 40.70 ± 10.30 | 40.56 ± 11.41 | 38.81 ± 9.30 | 0.19 (① vs. ②)  0.17 (② vs. ③) |
|  | 5^th^ day | 39.04 ± 10.92 | 40.34 ± 9.21 | 38.02 ± 7.66 |  |
| Cross-sectional  area of distal 1/3  flexors (cm^2^) | Baseline | 24.46 ± 5.43 | 24.45 ± 10.51 | 22.91 ± 4.85 | 0.07 (① vs. ②)  0.27 (② vs. ③) |
|  | 5^th^ day | 23.27 ± 6.49 | 22.43 ± 5.46 | 22.09 ± 3.93 |  |
| Total volume (cm^3^) | Baseline | 2396.13 ± 529.19 | 2478.28 ± 737.35 | 2351.70 ± 624.81 | 0.76 (① vs. ②)  0.37 (② vs .③) |
|  | 5^th^ day | 2286.57 ± 552.37 | 2417.87 ± 647.61 | 2400.26 ± 576.08 |  |

WF training with walkers on a floor; EEGR training with end-effector gait robot, Extensors include the quadriceps femoris; Flexors include the biceps femoris, semitendinosus and semimembranosus muscles, *p*-value by Mann-Whitney U tests or paired T-test.
