# Supplementary material for: Five-day rehabilitation of patients undergoing total knee arthroplasty using an end-effector gait robot as a neuromodulation blending tool for deafferentation, weight offloading and stereotyped movement: Interim analysis
Source: PLoS One. 2020 Dec 16;15(12):e0241117. doi: 10.1371/journal.pone.0241117 (PMC7743990; doi:10.1371/journal.pone.0241117)
Supplement: S2 Table — WF training with walkers on a floor; EEGR training with end-effector gait robot, EMG electromyography, VM vastus medialis, BF Biceps femoris; AUC area under the curve, p-value by two-way repeated measures analysis of variance or paired T-test. (DOCX) [file pone.0241117.s002.docx]

|  | | ①Operated knee in WF (n=5) | ②Operated knee in EEGR (n=9) | ③Non-operated knee in EEGR (n=9) | *p*-value |
| --- | --- | --- | --- | --- | --- |
| Peak torque of extensor (Nm) | Baseline | 12.10 ± 5.66 | 15.23 ± 8.04 | 77.03 ± 17.30 | 0.04 (① vs. ②)  0.08 (② vs. ③) |
|  | 5^th^ day | 18.98 ± 7.44 | 24.02 ± 13.56 | 89.77 ± 29.05 |  |
| Peak torque of extensor/body weight (Nm/kg) | Baseline | 21.34 ± 11.52 | 24.68 ± 11.62 | 126.38 ± 26.30 | 0.07 (① vs. ②)  0.06 (② vs. ③) |
|  | 5^th^ day | 32.71 ±10.06 | 36.51 ± 19.38 | 142.69 ± 42.41 |  |
| Maximal rate of torque development of extensor (Nm/s·kg) | Baseline | 0.14 ± 0.10 | 0.96 ± 2.27 | 0.96 ± 0.79 | 0.37 (① vs. ②)  0.41 (② vs. ③) |
|  | 5^th^ day | 0.16 ± 0.09 | 0.93 ± 0.28 | 1.00 ± 0.93 |  |
| Peak torque of flexor (Nm) | Baseline | 25.84 ± 7.97 | 31.37 ± 8.09 | 42.96 ± 11.03 | 0.03 (① vs. ②)  0.06 (② vs. ③) |
|  | 5^th^ day | 28.81 ± 8.36 | 37.97 ± 10.87 | 51.29 ± 16.36 |  |
| Peak torque of flexor/body weight (Nm/kg) | Baseline | 44.27 ± 17.37 | 51.82 ± 14.08 | 70.79 ± 16.94 | 0.04 (① vs. ②)  0.06 (② vs. ③) |
|  | 5^th^ day | 48.42 ± 12.15 | 62.50 ± 18.01 | 83.68 ± 23.80 |  |
| Maximal rate of torque development of flexor (Nm/s·kg) | Baseline | 0.11 ± 0.05 | 0.17 ± 0.09 | 0.30 ± 0.12 | 0.06 (① vs. ②)  0.07 (② vs. ③) |
|  | 5^th^ day | 0.15 ± 0.05 | 0.22 ± 0.10 | 0.36 ± 0.13 |  |
| Surface EMG peak amplitude of VM (mV) | Baseline | 839.61 ± 426.03 | 997.15 ± 710.53 | 3187.41 ± 1928.78 | 0.03 (① vs. ②)  0.06 (② vs. ③) |
|  | 5^th^ day | 922.12 ± 518.64 | 1859.16 ± 1690.14 | 4846.23 ± 1431.19 |  |
| Surface EMG peak amplitude of BF (mV) | Baseline | 1099.09 ± 799.48 | 1286.82 ± 832.18 | 3070.83 ± 1293.84 | 0.14 (① vs. ②)  0.13 (② vs. ③) |
|  | 5^th^ day | 1755.26 ± 1193.49 | 1864.92 ± 1278.02 | 3740.21 ± 2088.19 |  |
| Surface EMG mean amplitude of VM (mV) | Baseline | 181.75 ± 5.88 | 139.89 ± 3.59 | 159.49 ± 7.48 | 0.14 (① vs. ②)  0.16 (② vs. ③) |
|  | 5^th^ day | 197.59 ± 13.05 | 156.16 ± 4.62 | 174.00 ± 9.30 |  |
| Surface EMG mean amplitude of BF (mV) | Baseline | 207.58 ± 17.48 | 214.82 ± 41.48 | 723.73 ± 152.81 | 0.04 (① vs. ②)  0.06 (② vs. ③) |
|  | 5^th^ day | 211.95 ± 27.68 | 244.62 ± 25.09 | 785.31 ± 178.23 |  |
| Surface EMG total AUC of VM  (cm^2^/5 minutes) | Baseline | 413.59 ± 218.11 | 597.51 ± 394.44 | 1761.77 ± 1044.23 | 0.01 (① vs. ②)  0.06 (② vs. ③) |
|  | 5^th^ day | 617.03 ± 349.62 | 988.65 ± 664.45 | 3044.76 ± 900.35 |  |
| Surface EMG total AUC of BF  (cm^2^/5 minutes) | Baseline | 793.55 ± 825.64 | 788.15 ± 426.01 | 1902.60 ± 796.37 | 0.09 (① vs. ②)  0.06 (② vs. ③) |
|  | 5^th^ day | 1167.94 ± 848.44 | 1229.12 ± 818.05 | 2343.20 ± 983.10 |  |
| Surface EMG mean AUC of VM (cm/second) | Baseline | 21.58 ± 12.31 | 27.35 ± 11.34 | 62.79 ± 24.83 | 0.01 (① vs. ②)  0.06 (② vs. ③) |
|  | 5^th^ day | 27.05 ± 11.17 | 48.67 ± 24.36 | 104.76 ± 56.37 |  |
| Surface EMG mean AUC of BF (cm/second) | Baseline | 23.55 ± 15.34 | 28.15 ± 15.08 | 81.65 ± 36.93 | 0.09 (① vs. ②)  0.07 (② vs. ③) |
|  | 5^th^ day | 47.23 ± 25.45 | 49.58 ± 23.06 | 93.79 ± 83.14 |  |

WF training with walkers on a floor; EEGR training with end-effector gait robot, EMG electromyography, VM vastus medialis, BF Biceps femoris; AUC area under the curve, *p*-value by two-way repeated measures analysis of variance or paired T-test.
