# Supplementary material for: Five-day rehabilitation of patients undergoing total knee arthroplasty using an end-effector gait robot as a neuromodulation blending tool for deafferentation, weight offloading and stereotyped movement: Interim analysis
Source: PLoS One. 2020 Dec 16;15(12):e0241117. doi: 10.1371/journal.pone.0241117 (PMC7743990; doi:10.1371/journal.pone.0241117)
Supplement: S3 Table — WF training with walkers on a floor; EEGR training with end-effector gait robot; MEP motor evoked potential; BF biceps femoris; VM vastus medialis, H-reflex Hoffmann-reflex, p-value by two-way repeated measures analysis of variance or paired T-test. (DOCX) [file pone.0241117.s003.docx]

|  | | ①Operated knee in WF (n=5) | ②Operated knee in EEGR (n=9) | ③Non-operated knee in EEGR (n=9) | *p*-value |
| --- | --- | --- | --- | --- | --- |
| Mean amplitude of MEP of BF (mV) | Baseline | 0.82 ± 0.51 | 1.18 ± 0.91 | 1.43 ± 1.12 | 0.04 (① vs. ②)  0.06 (② vs. ③) |
|  | 5^th^ day | 1.20 ± 0.57 | 1.89 ± 1.62 | 2.33 ± 0.93 |  |
| Maximal amplitude of MEP of BF (mV) | Baseline | 1.04 ± 0.60 | 1.58 ± 1.09 | 1.94 ± 1.35 | 0.37 (① vs. ②)  0.27 (② vs. ③) |
|  | 5^th^ day | 2.06 ± 0.54 | 2.91 ± 5.18 | 3.17 ± 1.05 |  |
| Standardized amplitude of MEP of BF (mV/maximal M-wave) | Baseline | 0.78 ± 0.12 | 0.77 ± 0.19 | 0.74 ± 0.14 | 0.30 (① vs. ②)  0.29 (② vs. ③) |
|  | 5^th^ day | 0.87 ± 0.06 | 0.78 ± 0.28 | 0.84 ± 0.09 |  |
| Mean amplitude of MEP of VM (mV) | Baseline | 1.08 ± 1.04 | 0.97 ± 0.52 | 1.55 ± 1.07 | 0.01(① vs. ②)  0.06 (② vs. ③) |
|  | 5^th^ day | 1.26 ± 0.91 | 1.54 ± 0.92 | 2.42 ± 1.19 |  |
| Maximal amplitude of MEP of VM (mV) | Baseline | 1.30 ± 1.08 | 1.31 ± 0.73 | 2.16 ± 1.10 | 0.01(① vs. ②)  0.06 (② vs. ③) |
|  | 5^th^ day | 1.56 ± 0.89 | 1.86 ± 0.99 | 2.87 ± 1.37 |  |
| Standardized amplitude of MEP of VM (mV/maximal M-wave) | Baseline | 0.81 ± 0.19 | 0.78 ± 0.16 | 0.71 ± 0.16 | 0.14 (① vs. ②)  0.35 (② vs. ③) |
|  | 5^th^ day | 0.78 ± 0.19 | 0.81 ± 0.09 | 0.74 ± 0.13 |  |
| Maximal amplitude of H-reflex of BF (mV) | Baseline | 1.78 ± 0.34 | 1.59 ± 0.23 | 1.67 ± 0.31 | 0.04 (① vs. ②)  0.06 (② vs. ③) |
|  | 5^th^ day | 1.71 ± 0.41 | 1.67 ± 0.38 | 1.73 ± 0.32 |  |
| Standardized H-reflex of BF (mV/maximal M-wave) | Baseline | 0.39 ± 0.21 | 0.41 ± 0.11 | 0.31 ± 0.07 | 0.37 (① vs. ②)  0.56 (② vs. ③) |
|  | 5^th^ day | 0.38 ± 0.07 | 0.39 ± 0.07 | 0.28 ± 0.05 |  |
| Maximal amplitude of H-reflex of VM (mV) | Baseline | 0.42 ± 0.11 | 0.24 ± 0.11 | 0.45 ± 0.27 | 0.04 (① vs. ②)  0.06 (② vs. ③) |
|  | 5^th^ day | 0.38 ± 0.29 | 0.27 ± 0.12 | 0.52 ± 0.30 |  |
| Standardized H-reflex of VM (mV/maximal M-wave) | Baseline | 0.23 ± 0.10 | 0.26 ± 0.14 | 0.27 ± 0.14 | 0.43 (① vs. ②)  0.53 (② vs. ③) |
|  | 5^th^ day | 0.15 ± 0.11 | 0.15 ± 0.07 | 0.16 ± 0.08 |  |

WF training with walkers on a floor; EEGR training with end-effector gait robot; MEP motor evoked potential; BF biceps femoris; VM vastus medialis, H-reflex Hoffmann-reflex, *p*-value by two-way repeated measures analysis of variance or paired T-test.
