# Supplementary material for: Five-day rehabilitation of patients undergoing total knee arthroplasty using an end-effector gait robot as a neuromodulation blending tool for deafferentation, weight offloading and stereotyped movement: Interim analysis
Source: PLoS One. 2020 Dec 16;15(12):e0241117. doi: 10.1371/journal.pone.0241117 (PMC7743990; doi:10.1371/journal.pone.0241117)
Supplement: S4 Table — EMG electromyography; WF training with walkers on a floor; EEGR training with end-effector gait robot; VM vastus medialis, BF Biceps femoris, CMAP compound motor action potential; AUC area under the curve, p-value by Mann-Whitney U tests or paired T-test. (DOCX) [file pone.0241117.s004.docx]

| Surface EMG at post-interventional 3^rd^ weekday | | ①Operated knee in WF (n=5) | ②Operated knee in EEGR (n=9) | ③Non-operated knee in EEGR (n=9) | *p*-value |
| --- | --- | --- | --- | --- | --- |
| VM | Peak amplitude of CMAP (mV) | 5100.19 ± 2583.15 | 2059.44 ± 1435.54 | 2751.98 ± 1954.38 | 0.04 (① vs. ②)  0.06 (② vs. ③) |
|  | Mean amplitude of CMAP (mV) | 840.11 ± 284.59 | 774.56 ± 116.25 | 767.81 ± 112.83 | 0.69 (① vs. ②)  0.51 (② vs. ③) |
|  | Total area of AUC (cm^2^/5 minutes) | 67090.05 ± 8836.63 | 35008.25 ± 15984.85 | 58511.35 ± 27945.37 | 0.03 (① vs. ②)  0.06 (② vs. ③) |
|  | Mean area of AUC (cm/second) | 1241.74 ± 407.56 | 583.58 ± 266.47 | 975.38 ± 465.85 | 0.02 (① vs. ②)  0.06 (② vs. ③) |
| BF | Peak amplitude of CMAP (mV) | 2355.42 ± 52.030 | 2120.67 ± 1406.98 | 2084.91 ± 1020.34 | 0.51 (① vs. ②)  0.63 (② vs. ③) |
|  | Mean amplitude of CMAP (mV) | 759.89 ± 158.48 | 825.70 ± 155.90 | 813.87 ± 138.89 | 0.76 (① vs. ②)  0.83 (② vs. ③) |
|  | Total area of AUC (cm^2^/5 minutes) | 40682.46 ± 23919.24 | 38291.10 ± 19341.40 | 42106.84 ± 16462.51 | 0.46 (① vs. ②)  0.36 (② vs. ③) |
|  | Mean area of AUC (cm/second) | 686.50 ± 360.55 | 521.62 ± 322.42 | 701.92 ± 274.43 | 0.44 (① vs. ②)  0.53 (② vs. ③) |

EMG electromyography; WF training with walkers on a floor; EEGR training with end-effector gait robot; VM vastus medialis, BF Biceps femoris, CMAP compound motor action potential; AUC area under the curve, *p*-value by Mann-Whitney U tests or paired T-test.
