# Supplementary material for: Five-day rehabilitation of patients undergoing total knee arthroplasty using an end-effector gait robot as a neuromodulation blending tool for deafferentation, weight offloading and stereotyped movement: Interim analysis
Source: PLoS One. 2020 Dec 16;15(12):e0241117. doi: 10.1371/journal.pone.0241117 (PMC7743990; doi:10.1371/journal.pone.0241117)
Supplement: S1 Protocol — (PDF) [file pone.0241117.s005.pdf]

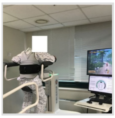

Five-day Rehabilitation of Patients Undergoing Total Knee Arthroplasty Using an End-Effector Gait Robot as a Neuromodulation Blending Tool for Deafferentation, Weight Offloading and Stereotyped Movement: Interim Analysis

Chang Ho Hwang[1]

[1]Department of Physical and Rehabilitation Medicine, Chungnam National University Sejoing Hospital, Chungnam National University College of Medicine, Sejong, Republic of Korea

dx.doi.org/10.17504/protocols.io.bfq5jmy6

Chang Ho Hwang

ABSTRACT

This prospective, randomized, single-blind controlled trial was performed at a tertiary medical center/university teaching hospital. The study protocol was registered atthe Protocol Registration and Results System (PRS), www.clinicaltrials.gov, (NCT02962453: https://clinicaltrials.gov/ct2/show/NCT02962453?term=NCT02962453&rank=1). The individual in this manuscript has given written informed consent to publish these case details.

The study included patients who had undergone unilateral TKA. Patients with central nervous system diseases, peripheral neuropathies, myopathies, or local muscle injuries around their thighs; patients who presented with any ambulatory impairments, decreased consciousness (Mini-Mental State Examination score < 23), or any unstable leg joints; patients with a history of arthroplasty of the legs or cardiac pacemaker implantation; and patients who refused to participate were excluded.

The enrolled patients were randomly allocated 1:1 to two groups in block-randomized order, as determined by a random assignment generator, with assignments delivered to one physical therapist in a sealed envelope. Instead of comparing patients with a sham group (only robot exposure with full weight bearing and without deafferentation), EEGR-assisted rehabilitation was compared with the gold standard of rehabilitation after TKA (ambulation training on a floor as early as possible). One group was subjected to 200-step rehabilitation using the EEGR, with a step length of 40 cm, a cadence of 40 cycles/minute, a speed of 0.98 km/hour, a guidance force of 100%, and a body weight support of 100%. The gait parameters of step length, cadence, and speed were selected after considering the postoperative state.

The second group was subjected to 200-step rehabilitation using walkers on a floor (WF); this treatment was conducted at a comfortable pace because the same speed as that of the 200-step rehabilitation protocol could not be guaranteed following TKA. Rehabilitation was performed three times per day for five consecutive weekdays. Measured data from the two groups were compared with respect to time (before and 5 days after the intervention).

All patients were provided identical physiotherapy, consisting of infrared radiation at a wavelength of 770 – 1500 *n*m, 45 cm from the patient's body, and perpendicular to the body surface, and continuous passive range of motion (ROM) exercises for 20 minutes.

The EEGR was equipped with a surrounding safety bar for manual grasping, a frontward chest support with a Velcro® strap to secure the trunk, a saddle for pelvic weight bearing, and a pair of end-effectors. The upper surface of the end-effectors completely contacted the patient's soles in the upright position and was persistently secured with ankle, mid-foot, and forefoot Velcro® straps, but free movement of the forefeet, ankles, and knees was allowed. An articulated robot arm with 3-degrees-of-freedom was located at each foot, and the robot arm had the footplate shape end-effector. The gait trajectory of the patient's feet was provided by the footplate. The patient's lower limb motion was guided along the foot movement trajectory. The robot had force sensors in the saddle and footplates. The sensors in the saddle could detect the upward or downward trajectory of the pelvis and adjust to its undulation to maintain the predetermined weight support and the sensors in the foot plates could detect ground reaction force on each foot in real-time during training. The amount of body weight support by the saddle and the amount of the ground reaction force on each foot plate were displayed on the monitor in real time. For example, in the case of 100% guidance, the entire weight of the patient is supported by the robot, and the whole movement of the legs is performed in full by the EEGR, the same method as that in Blicher *et al.*'s trial; on the monitor, the gauge for body weight support would display 100% of patient's weight and the ground reaction force gauges would indicate zero. For safety issues, if an excessive alteration in the symmetry between the feet or in the predetermined trajectory of the end-effectors on the ground reaction force was detected, the EEGR would be stopped instantly. A physiotherapist could also terminate its operation by pushing a button.

Started on May 06, 2020 01:24:26

This is an open access protocol distributed under the terms of the **Creative Commons Attribution License** (https://creativecommons.org/licenses/by/4.0/), which permits unrestricted use, distribution, and reproduction in any medium, provided the original author and source are credited

Finished steps: 0

Step 1 has not been completed

1

The study included patients who had undergone unilateral TKA. Patients with central nervous system diseases, peripheral neuropathies, myopathies, or local muscle injuries around their thighs; patients who presented with any ambulatory impairments, decreased consciousness (Mini-Mental State Examination score < 23), or any unstable leg joints; patients with a history of arthroplasty of the legs or cardiac pacemaker implantation; and patients who refused to participate were excluded.

Step 2 has not been completed

2 The enrolled patients were randomly allocated 1:1 to two groups in block-randomized order, as determined by a random assignment generator, with assignments delivered to one physical therapist in a sealed envelope. Instead of comparing patients with a sham group (only robot exposure with full weight bearing and without deafferentation), EEGR-assisted rehabilitation was compared with the gold standard of rehabilitation after TKA (ambulation training on a floor as early as possible). One group was subjected to 200-step rehabilitation using the EEGR, with a step length of 40 cm, a cadence of 40 cycles/minute, a speed of 0.98 km/hour, a guidance force of 100%, and a body weight support of 100%. The gait parameters of step length, cadence, and speed were selected after considering the postoperative state.

The second group was subjected to 200-step rehabilitation using walkers on a floor (WF); this treatment was conducted at a comfortable pace because the same speed as that of the 200-step rehabilitation protocol could not be guaranteed following TKA. Rehabilitation was performed three times per day for five consecutive weekdays. Measured data from the two groups were compared with respect to time (before and 5 days after the intervention).

All patients were provided identical physiotherapy, consisting of infrared radiation at a wavelength of 770 – 1500 nm, 45 cm from the patient's body, and perpendicular to the body surface, and continuous passive range of motion (ROM) exercises for 20 minutes.

Step 3 has not been completed

3 The EEGR was equipped with a surrounding safety bar for manual grasping, a frontward chest support with a Velcro® strap to secure the trunk, a saddle for pelvic weight bearing, and a pair of end-effectors. The upper surface of the end-effectors completely contacted the patient's soles in the upright position and was persistently secured with ankle, mid-foot, and forefoot Velcro® straps, but free movement of the forefeet, ankles, and knees was allowed. An articulated robot arm with 3-degrees-of-freedom was located at each foot, and the robot arm had the footplate shape end-effector. The gait trajectory of the patient's feet was provided by the footplate. The patient's lower limb motion was guided along the foot movement trajectory. The robot had force sensors in the saddle and footplates. The sensors in the saddle could detect the upward or downward trajectory of the pelvis and adjust to its undulation to maintain the predetermined weight support and the sensors in the foot plates could detect ground reaction force on each foot in real-time during training. The amount of body weight support by the saddle and the amount of the ground reaction force on each foot plate were displayed on the monitor in real time. For example, in the case of 100% guidance, the entire weight of the patient is supported by the robot, and the whole movement of the legs is performed in full by the EEGR, the same method as that in Blicher et al.'s trial; on the monitor, the gauge for body weight support would display 100% of patient's weight and the ground reaction force gauges would indicate zero. For safety issues, if an excessive alteration in the symmetry between the feet or in the predetermined trajectory of the end-effectors on the ground reaction force was detected, the EEGR would be stopped instantly. A physiotherapist could also terminate its operation by pushing a button.

This is an open access protocol distributed under the terms of the **Creative Commons Attribution License** (https://creativecommons.org/licenses/by/4.0/), which permits unrestricted use, distribution, and reproduction in any medium, provided the original author and source are credited
